# Cue-Elicited Craving in Heroin Addicts at Different Abstinent Time: An fMRI Pilot Study

**DOI:** 10.3109/10826084.2011.646381

**Published:** 2012-02-13

**Authors:** Mingwu Lou, Erlei Wang, Yunxia Shen, Jiping Wang

**Affiliations:** 1 Department of Radiology, Longgang Central Hospital, Shenzhen, China; 2 Department of Graduate, Guangzhou University of Chinese Medicine, Guangzhou, China; 3 Department of Radiology, The First Hospital of JiLin University, Changchun, China

**Keywords:** abstinence, cue-reactivity, craving, heroin dependence, fMRI

## Abstract

*Objective:* We evaluated the effect of short-term and long-term heroin abstinence on brain responses to heroin-related cues using functional magnetic resonance imaging (fMRI). *Methods:* Eighteen male heroin addicts following short-term abstinence and 19 male heroin addicts following long-term abstinence underwent fMRI scanning while viewing heroin-related and neutral images. Cue-elicited craving and withdrawal symptoms in the subjects were measured. *Results:* Following short-term abstinence, greater activation was found in response to heroin cues compared to neutral cues in bilateral temporal, occipital, posterior cingulate, anterior cingulate, thalamus, cerebellum, and left hippocampus. In contrast, activations in bilateral temporal and occipital and deactivations in bilateral frontal, bilateral parietal, left posterior cingulate, insula, thalamus, dorsal striatum, and bilateral cerebellum were observed following long-term abstinence. Direct comparisons between conditions showed greater brain reactivity in response to smoking cues following short-term abstinence. In addition, short-term abstinence had more serious withdrawal symptoms than the long-term. *Conclusion:* The present findings indicate that compared to short-term, long-term abstinence manifests less serious withdrawal symptoms and significantly decreases neural responses to heroin-related cues in brain regions subserving visual sensory processing, attention, memory, and action planning. These findings suggest that long-term abstinence can decrease the salience of conditioned cues, thereby reducing the risk of relapses. The study's limitations are noted.

## RÉSUMÉ

**Cue-Craving suscité chez les héroïnomanes à différents Temps d'abstinence: une étude IRMf**

*Objectif:* Nous avons évalué l'effet de l'abstinence d'héroïne à court terme et à long terme sur les réponses du cerveau à l'héroi'ne signaux liés à l'aide de l'imagerie par résonance magnétique fonctionnelle (IRMf). *Méthodes:* Dix-huit héroïnomanes mâles après l'abstinence à court terme et dix-neuf héroïnomanes mâles après abstinence à long terme ont subi de balayage IRMf pendant la visualisation liés à l'héroïne et des images neutres. Cue-a suscité les symptômes de manque et de retrait dans les sujets ont été mesurés. *Résultats:* Après l'abstinence à court terme, une plus grande activation à été trouvée en réponse à l'héroïne par rapport à des indices des indices neutres dans bilatéraux temporelle, occipital, cingulaire postérieur, cingulaire antérieur, l'hippocampe thalamus, le cervelet et la gauche. En revanche, les activations dans les accords bilatéraux désactivations temporelle, occipital et dans les accords bilatéraux frontal, pariétal bilatéral, cingulaire postérieur gauche, l'insula, le thalamus, le striatum dorsal et le cervelet bilatérales ont été observées après abstinence à long terme. Les comparaisons directes entre les conditions ont montré une réactivité plus grande du cerveau en réponse à des signaux de fumer à court terme suivantes abstinence. De plus, à court terme l'abstinence avait des symptômes de sevrage plus graves que le long terme. *Conclusion:* Les résultats actuels indiquent que, comparativement à court terme, abstinence à long terme à des symptômes de sevrage moins graves et diminue considérablement les réponses neurales à l'héroïne signaux liés dans les régions cérébrales visuelles subserving traitement sensoriel, l'attention, la mémoire et la planification des actions. Ces résultats suggèrent que l'abstinence à long terme peut diminuer la saillance des indices conditionné, réduisant ainsi le risque de rechute.

## Abstract (es)

**Cue-suscitó el deseo en adictos à la heroína en diferentes La abstinencia de tiempo: un estudio de fMRI**

*Objetivo:* Se evaluó el efecto de la abstinencia de heroína a corto plazo y largo plazo en las respuestas del cerebro a la heroína relacionadas con el uso de las señales de resonancia magnética funcional (fMRI). *Métodos:* Dieciocho hombres adictos a la heroína a corto plazo que la abstinencia y diecinueve hombres adictos a la heroína después de abstinencia a largo plazo se sometieron a exploratión fMRI mientras se ve relacionada con la heroína y las imágenes neutras. Cue-suscitó el deseo y los síntomas de abstinencia en los sujetos fueron medidos. *Resultados:* Después de la abstinencia a corto plazo, una mayor activatión se encuentra en respuesta a las señales de heroína en comparación con las señales neutrales bilateral temporal, cíngulo occipital, posterior, anterior cingulada hipocampo, el tálamo, el cerebelo y se fue. En contraste, las activaciones de los acuerdos bilaterales desactivación temporal, occipital y en los acuerdos bilaterales frontal, parietal bilateral, posterior izquierdo ínsula cingulada,, tálamo, cuerpo estriado dorsal y el cerebelo bilateral se observaron tras abstinencia a largo plazo. Las comparaciones directas entre las condiciones mostearon una mayor reactividad del cerebro en respuesta a las señales de fumar a corto plazo que la abstinencia. Además, a corto plazo la abstinencia tenían síntomas de abstinencia más grave que la de largo plazo. *Conclusión:* Los resultados actuales indican que en comparación con el corto plazo, a largo plazo la abstinencia tiene síntomas de abstinencia menos graves y reduce significativamente las respuestas neuronales a la heroína relacionadas con las señales en las regiones cerebrales subserving procesamiento sensorial visual, atención, memoria y planificación de la actión. Estos hallazgos sugieren que la abstinencia a largo plazo puede disminuir la relevancia de las señales acondicionado, reduciendo así el riesgo de recaídas.

## INTRODUCTION

Drug addiction is characterized by compulsive drug-taking behavior and high rates of relapse even after many years of abstinence (O'Brien, Testa, O'Brien, Brady, & Wells, 1977). Exposure to cues associated with drug use instigates physiological, behavioral, and subjective reactions, including craving, and is thought to play a significant role in the maintenance of the addiction, as well as the relapse in people attempting to quit (Abrams, Monti, Carey, Pinto, & Jacobus, 1988). Over the past few decades, numerous functional magnetic resonance imaging (fMRI) studies have documented brain areas activated by drug-related cues. These areas include regions known to be involved with reward, craving, emotional processing, memory, visual attention, and impulsivity (Brody et al, 2007; Due, Huettel, Hall, & Rubin, 2002; Franklin et al., 2007; McClernon, Kozink, Lutz, & Rose, 2009; Rubinstein et al., 2011). These findings suggest that exposure to drug-related cues increases attentional resources focused on processing external, drug-related information, and triggers the planning of behaviors aimed at obtaining drugs.

However, one factor that is believed to influence this neural response to drug cues – the user's level of abstinence – has seldom been investigated using functional brain imaging. Brain reactivity to smoking-related cues increases following acute and extended smoking abstinence compared to the prequit state (Janes et al., 2009; McClernon et al., 2009). In contrast with these findings, two previous fMRI studies did not show that smoking abstinence increased brain reactivity to smoking cues (McClernon, Hiott, Huettel, & Rose, 2005; McBride, Barrett, Kelly, Aw, & Dagher, 2006), while one study found abstinence did decrease reactivity in ventral striatum in male smokers (David et al., 2007).

Collectively, the above studies all evaluated brain activity in response to smoking-related versus neutral images in tobacco-dependent people before a quit attempt and then again during acute or extended smoking. Abstinence on brain responses to heroin-related cues in people who abuse heroin has seldom been studied. Therefore, the primary goal of the present study was to evaluate the effects of short- and long-term heroin abstinence on neural responses to heroin-related visual cues in heroin addicts, and to elucidate mechanisms underlying the effect of heroin abstinence on heroin-related cue reactivity, which is critical since exposure to these cues can trigger heroin relapse (Kushnir et al, 2010; Mei, Zhang, & Xiao, 2010; Nestor, McCabe, Jones, Clancy, & Garavan, 2011).

## MATERIALS AND METHODS

### Subjects

Eighteen heroin addicts during short-term abstinence and 19 heroin addicts during long-term abstinence were recruited. All subjects were receiving inpatient treatment in the Forced Detoxification Center of Longgang Central Hospital, Shenzhen, China, where they were prescreened and interviewed by the treatment staff and forced to accept treatment for about two years and were not able to use heroin. In our study, subjects were included if they met following criteria: (1) male, (2) age 18–55 years (note the age range), (3) right handed, (4) 20/20 vision, (5) diagnosed with DSM-IV heroin dependence, and (6) heroin was their primary drug of use and no current psychotropic medication was being used. For safety reasons, heroin addicts with magnetically active prosthetics, plates, pins, permanent retainers, or bullets were excluded. Patients, who voluntarily participated in this study, had the right to quit the experiments at anytime. All patients who finished the study received monetary compensation.

Data of two subjects who had long-term heroin abstinence were excluded because of excessive head movement or incomplete fMRI data. Data of one subject who had short-term heroin abstinence were excluded for incomplete fMRI data due to technical failures. As a consequence, the final data were collected from 17 heroin-dependent subjects who were short-term abstainers and 17 heroin-dependent subjects who were long-term abstainers. The demographic and substance-use characteristics of the subjects are summarized in Table 1.

**TABLE 1:** Demograpic and clinical characteristics of subjects

Characteristic	Short-term heroin abstinence (*n* = 18)	Long-term heroin abstinence (*n* = 19)	*p*-value from ANOVA
Age (years ± SD)	33.2 ± 1.4	31.6 ± 1.4	0.415
Education (years ± SD)	7.7 ± 0.7	8.1 ± 0.4	0.662
Duration of heroin use (years ± SD)	7.0 ± 1.0	8.2 ± 1.1	0.430
Average heroin dose (g/day ± SD)	0.7 ± 0.2	0.7 ± 0.1	0.818
Duration of current abstinence (months ± SD)	1.2 ± 0.1	13.6 ± 0.4	0.000
Duration of smoking (years ± SD)	12.9 ± 1.4	13.8 ± 1.2	0.632
Daily cigarettes	32.4 ± 3.5	29.8 ± 2.7	0.561
Depression	13.1 ± 1.5	9.8 ± 1.7	0.158
Anxiety	16.3 ± 2.5	10.7 ± 2.2	0.100
Withdrawal symptoms	23.5 ± 3.4	11.6 ± 1.8	0.004

The only difference between the forced and the self-initiated detoxification treatment in China is that the forced detoxification subjects are sent to the detoxification center by the law enforcement officials while the self-initiated detoxification subjects voluntarily go to the detoxification center or are escorted to the center by their family members. The following detoxification procedures are the same for these two groups: they will receive medication and psychological therapy at the center; they are strictly restricted to drugs; they will stay up to two years at the center; afterwards, they will be released but will be closely followed up.

### Procedures

All subjects completed two sessions: a half-an-hour screening/practice session and a 1-h fMRI session. At the beginning of the screening/practice session, subjects heard a complete description of the study, and read and signed an informed consent form approved by the Institutional Review Board of Longgang Central Hospital of Shenzhen. They then completed questionnaires regarding their health, mood, smoking history, and eligibility for fMRI research. They also practiced an experimental task in a mock fMRI scanner.

All scanning was conducted between 15:00 to 18:00 PM. Subjects were escorted to the scanning facility and placed on the magnetic resonance imaging (MRI) scanner bed with their head secured using a vacuum bag to minimize movement. Earplugs were used to lessen scanner noise. Then sets of MRI images were acquired to provide the anatomical information about the brain. All subjects were monetarily compensated upon completion of the study.

### Preliminary Behavioral Assay

Another 30 heroin addicts were selected at the Forced Detoxification Center of Longgang Central Hospital, Shenzhen, China. Fifty pictures of heroin-related cues and 50 pictures of neutral cues were shown on a computer to these subjects. Craving of each subject was assessed on a 10-point visual analog scale (VAS). Participants marked 1 for “not at all” to 10 for “extremely high” based on their true response to each picture. As predicted, heroin-related cues evoked stronger craving in these subjects than neutral cues. We finally used 40 pictures each for our test.

### Cue-Viewing Task

**FIGURE 1:**
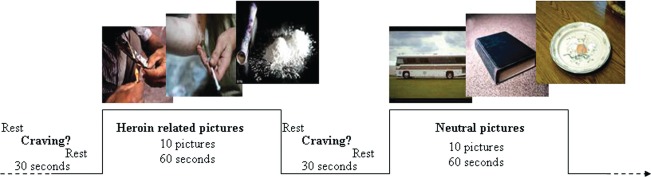
Experimental design.

Photographic heroin-related and neutral cues were presented in a boxcar with four blocks. Each block contained a cue of a certain category, as shown in Figure 1. Images were shown on a computer screen and reflected by an overhead mirror through which the subjects could see the cues. Heroin-related cues (*n* = 40) consisted of pictures of people using heroin. Neutral cues (*n* = 40) consisted of pictures of people engaged in everyday activities. Prior to this study, those heroin-related cues had been proved to evoke significant craving in heroin addicts by our preliminary behavioral assay. Ten cues in each block were shown in 1 min (McClernon et al, 2009). Before and after each block, a crosshair was presented for 5 s. Participants were then asked to rate their current craving level on a four-point scale (with “not at all” = 0 to “extremely” = 4). The scale was presented for 10 s followed by a crosshair for another 15 s. Thus, the total interblock-interval was 30 s. Total task time was 12.5 min. Stimulus presentations were delivered by using the E-Prime software package (Psychology Software Tools, Inc., Pittsburgh, PA, USA). The timing of the stimulus presentation was synchronized with trigger pulses from the MRI scanner to ensure precise temporal integration of stimulus presentation and fMRI data acquisition. The scale selected by each subject was recorded.

### fMRI Acquisition

Scanning was performed on a 1.5-T Philips Infinion MR system (Philips Medical Systems, Netherlands) at Longgang Central Hospital. For the functional run, 250 volumes, each with 24 axial slices, were obtained with a T2-weighted echo-planar imaging (EPI) sequence (TR = 3 s, TE = 40 ms, FOV = 24 × 24 cm, matrix = 64 × 64, flip angle = 90°, slice thickness = 4 mm, gap = 2 mm). A coplanar T1-weighted structural volume was obtained with a spin echo (SE) sequence (matrix = 256 × 256). A high-resolution structural volume was obtained with a spoiled gradient recalled echo (SPGR) sequence for functional overlay.

### Data Analysis

Demographic and behavioral data were analyzed using SPSS software (Version 17.0.1 for Windows; SPSS Inc., Chicago, IL). Imaging data were analyzed using SPM5 (Statistical Parametric Mapping; Wellcome Department of Cognitive Neurology, London, UK). The first four volumes of each functional time series were discarded for saturation effects. Images were re-oriented and re-aligned to the first volume to correct for between-scan movements. The anatomical scan was coregistered to the first T2* image. Next, the images were normalized to Montreal Neurological Institute (MNI) space using an SPM EPI template (using 12 linear parameters and a set of nonlinear cosine basis functions). Spatial smoothing was performed using a 4-mm full-width-at-half-maximum Gaussian kernel.

Each participant's data was used for a first-level voxel-by-voxel analysis using the general linear model. Each cue block (heroin-related, natural) was modeled as a boxcar function convolved with a canonical hemodynamic response function that began at the onset of the first cue in the block and ended at the end of the block (duration = 60 s). A high-pass filter (1/128 Hz) was applied to remove slow signal drift. A contrast image was generated by computing the difference between a heroin-related cue and a neutral cue. One-sample *t*-tests were performed to examine heroin-related cue reactivity for each condition, respectively (short-term heroin abstinence, long-term heroin abstinence). Two-sample *t*-test was conducted to examine the differences in brain responses between those two conditions. Resulted activations were considered significant at a *p* ≤ 0.05 (family-wise error corrected) with a minimum cluster extent threshold of 20 contiguous voxels.

### Withdrawal Symptoms Measures

The withdrawal symptoms measures included three components: somatization, negative mood, and dyssomnia. Each component was assessed by a separate questionnaire. The somatization questionnaire consisted of eight questions, rated on a scale of 0 (no effect) to 4 (strongest). The questionnaire asked about the following symptoms: (1) panic, (2) bodily malaise, (3) restlessness, (4) musculoskeletal pain, (5) sluggishness, (6) yawning, tearing, and runny nose, (7) gooseflesh, and (8) loss of appetite. The negative-mood questionnaire consisted of four questions, rated on a scale of 0 (no effect) to 4 (strongest). The questionnaire asked about the following symptoms: (1) dysphoria, (2) loneliness, (3) loss of interest in daily activities, and (4) irritability. The dyssomnia questionnaire consisted of five questions, rated on a scale of 0 (no effect) to 4 (strongest). The questionnaire asked about the following symptoms: (1) insufficient sleep duration, (2) difficulty falling asleep, (3) restless sleep, (4) early awakening, and (5) dizziness on awakening (Shi, Zhao, Epstein, Zhang, & Lu, 2007).

## RESULTS

### Subject Characteristics

As shown in Table 1, there were no significant differences between groups on demographic measures. The abstinence was assessed using urine test and physical symptoms. All drug user patients were only opioid users.

### Protracted-Abstinence Symptoms

In this study, we observed significant difference in protracted-abstinence symptoms between groups. The group during long-term abstinence had decreased protracted-abstinence symptoms than the group during short-term abstinence.

### Craving Scores

Across conditions, craving for cigarettes was greater following heroin-related cues compared to neutral cues. Moreover, across cue types, abstinence increased ratings of craving during the cue-viewing task. The “Cue × Condition” interaction was not significant.

### Imaging Results

**FIGURE 2:**
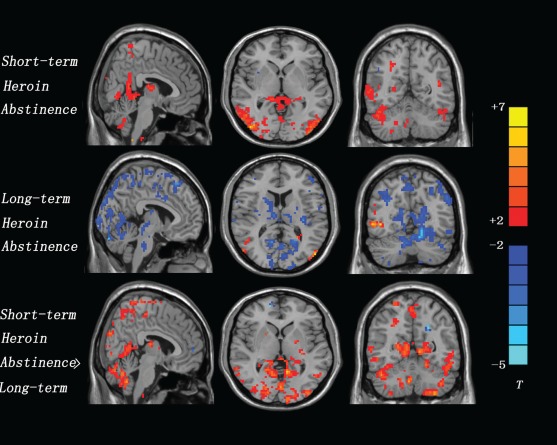
A representative scanning result from a patient.

The effect of heroin-related cues (compared to neutral cues) on brain activation was evaluated separately for each condition. Following short-term abstinence, heroin addicts exhibited significantly greater fMRI reactivity to heroin-related versus neutral images in a number of brain areas. These included bilateral temporal (BA 19, 21, 39), bilateral occipital (BA 19, 37, 39), bilateral posterior cingulate (BA 23, 29, 30), bilateral anterior cingulate, bilateral thalamus, left hippocampus, and bilateral cerebellum. The reduced fMRI reactivity to heroin-related images versus neutral images was not found in any brain areas (Figure 2; Table 2).

**TABLE 2:** fMRI measurement following short-term heroin abstinence

				Talairach coordinates			
Side	Brain area	BA	Cluster size (mm^3^)	*x*	*y*	*z*	Voxels
*Occipital cortex*							
R	Inferior occipital G.	19	136	33	–78	–15	3.2
L	Middle occipital G.	19, 37, 39	176	–45	–87	3	7.3
R	Middle occipital G.	19, 37, 39	236	45	–84	0	8.7
L	Fusiform G.	37	60				
R	Fusiform G.	37	64	36	–60	–18	4.4
L	Cuneus	17	55	–12	–87	9	3.1
R	Cuneus	17	67	21	–81	9	3.9
*Temporal cortex*							
L	Middle temporal G.	21,22	52	–60	–69	12	3.3
R	Middle temporal G.	21, 22, 39	149	57	–57	3	3.9
R	Inferior temporal G.	20	109	54	–69	–6	4.9
L	Parahippocampal G.	35	102	–17	–21	–18	5.1
*Cingulate*							
	Posterior	23	89	–3	–51	12	5.2
	Anterior	33	22	–3	9	27	3.9
*Thalamus*							
L	Thalamus		20	0	–18	12	3.6
R	Thalamus		11	3	–15	9	3.6
*Hippocampus*							
L	Hippocampus		60				
*Cerebellum*							
L	Cerebellum		102	–33	–72	–24	2.9
R	Cerebellum		242	42	–63	–21	3.7

*Note.* Brain areas, Brodmann areas, and Talairach coordinates (Talairach & Tournoux, 1988) refer to the peak activation voxel within each cluster of continuous voxels. The *t*–values refer to the maximum *t* statistic in each cluster. Voxel numbers refer to the total number of voxels per cluster. G = gyrus.

Following long-term abstinence, heroin addicts exhibited greater fMRI reactivity to the heroin-related than to the neutral images in bilateral temporal (BA 21) and bilateral occipital (BA 19). The reduced fMRI reactivity to heroin-related images versus the neutral images was evoked in bilateral frontal (BA 6, 8, 9), bilateral parietal (BA 1, 2, 3, 5, 7,40), left posterior cingulate (BA 23), in-sula, thalamus, dorsal striatum, and bilateral cerebellum (Figure 2; Table 3).

**TABLE 3:** fMRI measurement following long-term heroin abstinence

				Talairach coordinates			
Side	Brain area	BA	Cluster size (mm^3^)	*x*	*y*	*z*	Voxels
Heroin-related > Neutral Images Brain Area							
*Occipital cortex*							
L	Inferior occipital G.	19	15	36	–81	–12	2.6
R	Inferior occipital G.	19	19	–24	–93	–6	2.5
L	Middle occipital G.	19	55	–33	–87	3	2.7
R	Middle occipital G.	19	40	42	–63	–3	8.7
*Temporal cortex*							
L	Middle temporal G.	21,22	15	–42	–78	6	2.4
R	Middle temporal G.	21,22	47	54	–75	6	3.4
L	Inferior temporal G.	37	11	–51	–78	–6	2.9
R	Inferior temporal G.	37	19	48	–66	–3	3.6
Neutral > Heroin-related Images Brain Area							
*Frontal cortex*							
L	Middle frontal G.	6,9	206	–39	15	33	–5.2
L	Superior frontal G.	8	66	–3	24	54	–4.9
L	Inferior frontal G.	46	17	–45	48	6	–4.7
*Parietal cortex*							
L	Inferior parietal G.	40	425	–39	–48	57	–2.4
L	Precuneus	7	167	–6	–45	54	–3.9
R	Precuneus	7	198	12	–48	66	–7.2
L	Paracentral lobule	5	133	–3	–33	51	–5.0
R	Postcentral G.	1,2,3	144	45	–30	57	–4.4
*Cingulate*							
L	Posterior cingulate		140	–3	–66	12	–3.4
*Thalamus*							
R	Thalamus		37	9	–18	12	–4.1
*Insula*							
L	Insula	13	45	–33	–24	12	–4.7
*Striatum*							
L	Lentiform nucleus		13				
R	Lentiform nucleus		21				
L	Putamen		13				
R	Putamen		21				
*Cerebellum*							
L	Cerebellum		102	–33	–72	–24	2.9
R	Cerebellum		242	42	–63	–21	3.7

When fMRI activity to heroin-related versus neutral images was compared across conditions, several brain areas were found to exhibit increased activation following the short-term abstinence in comparison to the long-term abstinence. Increased activation was found in the bilateral temporal (BA 19, 21, 39), bilateral parietal (BA 1, 2), bilateral occipital (BA 19, 37, 39), left posterior cingulate (BA 23, 29, 30), left hippocampus, insula, thalamus, dorsal striatum, and bilateral cerebellum. The reduced fMRI reactivity to heroin-related images versus neutral images was not found in any brain areas (Figure 2, Table 4).

**TABLE 4:** Between-scan fMRI reactivity differences: (Heroin-Related > Neutral Images)

				Talairach coordinates			
Side	Brain area	BA	Cluster size (mm^3^)	*x*	*y*	*z*	Voxels
*Occipital cortex*							
L	Middle occipital G.	19,37,39	190	–27	–96	6	5.2
L	Lingual G.	17	167	–15	–63	–9	4.0
R	Lingual G.	17	150	18	–90	0	3.5
L	Cuneus		316	–18	–96	6	5.2
*Temporal cortex*							
L	Middle temporal G.	21,22	43	–51	–60	0	2.6
L	Parahippocampal G.	35	118	–27	–69	–15	3.5
L	Fusiform G.	19	185				
R	Fusiform G.	19	51				
L	Inferior temporal G.		92				
R	Inferior temporal G.		56				
*Parietal cortex*							
L	Precuneus		97				
R	Precuneus		90	12	–63	15	3.5
L	Paracentral		61	–6	–36	0	2.7
*Cingulate*							
	Posterior	23	174	–3	–63	12	3.1
	Anterior		8	23	9	26	3.5
*Thalamus*							
L	Thalamus		15				
R	Thalamus		7	15	–9	15	3.6
*Insula*							
	Insula	13	10	–33	–21	12	3.5
*Hippocampus*							
L	Hippocampus		60	21	12	15	3.2
*Striatum*							
L	Putamen		7				
R	Putamen		8				
L	Pallidum		11				
*Cerebellum*							
L	Cerebellum		102	–33	–72	–24	2.9
R	Cerebellum		242	42	–63	–21	3.7

## DISCUSSION

This study was a pilot study. We used fMRI to detect brain activity in response to heroin-related versus neutral images in heroin addicts following short- and long-term abstinence, respectively.

We found remarkable between-scan differences in fMRI reactivity patterns between the two groups. Following short-term heroin abstinence, fMRI reactivity to heroin-related versus neutral images increased in regions involved in visuospatial processing such as occipital cortex (McClernon, Kozink, & Rose, 2008; Thiel, Zilles, & Fink, 2005). Similar sites of activation have been observed in previous studies of smoking cue reactivity (Brody et al, 2007; McBride et al., 2006; Wilson, Sayette, Delgado, & Fiez, 2005). Greater heroin-related cue activation was also observed in temporal areas that are correlated with increased reactivity to heroin-related cues (McClernon et al., 2008). Heroin-related cue activation increased in posterior cingulate, which is involved in visuospatial attention and information processing (Gron, Wunder-lich, Spitzer, Tomczak, & Riepe, 2000; Vogt, Finch, & Olson, 1992) and has been shown active in response to visual smoking (McBride et al., 2006) and cocaine cues (Kilts, Gross, Ely, & Drexler, 2004). Heroin-related cue activation was also observed in anterior cingulate, which is involved in emotional processing (Fichtenholtz et al., 2004; Keightley et al., 2003). Heroin-related cues activated hippocampus and thalamus in addition to the aforementioned areas of the brain. The hippocampus has an important role in learning, memory, and anxiety (Buccafusco et al., 1995). Following long-term heroin abstinence, greater heroin-related cue activation was only observed in occipital and temporal cortices.

Strong activities were evoked in dorsal striatum by the heroin-related cues following short-term abstinence. The dorsal striatum plays a predominant role in the maintenance of drug-seeking behavior (Haber, Fudge, & McFarland, 2000; Ito, Dalley, Robbins, & Everitt, 2002). Microinjection of a dopamine receptor or AMPA/kainite glutamate receptor antagonist directly into the dorsal striatum attenuated cocaine seeking maintained under a second-order schedule of reinforcement (Belin & Everitt, 2008; Vanderschuren, Di Ciano, & Everitt, 2005). Inactivation of the dorsal striatum was shown to reduce relapse to cocaine seeking driven by discrete or contextual cues (Fuchs, Branham, & See, 2006; See, Elliott, & Feltenstein, 2007). Studies in humans have shown increases in activity and dopamine transmission during cue-induced cocaine craving in the dorsal, but not ventral, striatum (Garavan et al., 2000; Volkow et al., 2006; Wong et al., 2006). We have demonstrated that decreased neural responding to heroin-related cues in dorsal striatum may reflect decreased relapse vulnerability during long-term abstinence.

Decreased activities were evoked in the insula by the heroin-related cues following long-term abstinence. The insula is reportedly involved in maintaining smoking behavior (Naqvi, Rudrauf, Damasio, & Bechara, 2007), and it is activated during various forms of craving and/or exposure to drug-related or other incentive-related cues (Craig, Attwood, Benton, Penton-Voak, & Munafo, 2009). Our data suggest that long-term heroin abstinence can abolish cue-associated interoceptive sensations mediated by the insula, which may decrease craving responses.

Together, our data suggest that a number of brain areas exhibit decreased reactivity in comparison to the short-term abstinence state, during long-term heroin abstinence. These brain areas may facilitate craving and habitual responding to smoking cues and predispose people to relapse, and may be useful targets for relapse prevention medications.

### Study's Limitations

There were several limitations in this study. First, as with many other studies, this study had limited sample size and we had to use a between-subjects design. Although we tried our best to minimize the individual difference in terms of age and the amount and length of heroin intake, the craving behavior among individual subjects were different. Second, although all the subjects in this study were free to take part in many activities in the clinic, they were confined to drug access through hospitalization, which inevitably caused some stress in these subjects. The stress might have some impact on our results. Therefore, further studies with outpatients are needed to cross-validate our findings.

Notwithstanding these limitations, our results indicate that compared with those following short-term abstinence, male heroin addicts following long-term abstinence exhibit smaller fMRI reactivity in the dorsal caudate nucleus and other brain areas involved in learning, action planning, and motor behavior, which may contribute to relapse vulnerability.
